# Canagliflozin treatment of a horse with hyperinsulinemia, including pharmacokinetic analysis of different dosing protocols: a case report

**DOI:** 10.3389/fvets.2026.1838222

**Published:** 2026-07-13

**Authors:** Cecily Burbidge, Katelyn White, Kate Armstrong, Brielle Rosa

**Affiliations:** Faculty of Veterinary Medicine, University of Calgary, Calgary, AB, Canada

**Keywords:** canagliflozin, equine, glucose, insulin dysregulation, laminitis, triglycerides

## Abstract

Laminitis is a complex and painful disease of the equine foot. Insulin dysregulation (ID) resulting in hyperinsulinemia is responsible for more than 90% of cases in the general horse and pony population. Sodium-glucose cotransporter 2 inhibitors (SGLT2i) are a relatively new class of drug that has gained considerable popularity as an off-label treatment of hyperinsulinemia-associated laminitis (HAL) in horses when traditional management changes and treatments are unsuccessful. In Canada, canagliflozin is the SGLT2i most available to equine veterinarians; although no dosing protocols have been well-validated in horses, it is commonly administered orally at 0.6 mg/kg once daily. However, due to its long half-life in the horse, we hypothesized that every other day (EOD) dosing might be efficacious. Here we report the clinical outcome and pharmacokinetics of once daily versus EOD canagliflozin in a teaching horse with hyperinsulinemia and a history of severe laminitis. We found that insulin levels in this patient could be maintained within normal limits using EOD oral treatment of canagliflozin at a dose of 0.6 mg/kg. Daily dosing of canagliflozin led to accumulation resulting in approximately three times the drug exposure achieved with EOD dosing, where no accumulation was observed. Maximum plasma concentrations were 934.2 ng/mL after 30 days of single daily dosing and 324.3 ng/mL after 30 days of EOD dosing. This may be a promising new dosing strategy, although prospective trials in risk-matched populations are needed to assess the incidence of medication-associated adverse events and laminitis to determine the safety profiles of canagliflozin dosing strategies.

## Introduction

Hyperinsulinemia puts horses at risk of laminitis, a painful and debilitating condition of the equine foot (1, 2). Traditionally, management has focused on reducing dietary simple carbohydrate intake to limit pancreatic insulin secretion. In some cases, concurrent conditions, such as pituitary pars intermedia dysfunction, may also contribute to hyperinsulinemia and warrant separate management (3). Despite treatment, a subset of horses will have persistently high blood insulin concentrations, and repeated, or severe, episodes of laminitis can be career- or life-ending for them (4). Pharmaceutical treatments that have previously been tried for these refractory cases include the anti-diabetes drug metformin, which is used to regulate blood sugar in humans with Type 2 diabetes. However, this medication has been reported to have poor (~7%) bioavailability in horses (5) and is rarely successful at controlling hyperinsulinemia (6) despite some reports of modest clinical benefits in ID horses (7). Levothyroxine, a synthetic thyroid hormone, has also been used to manage hyperinsulinemia by increasing basal metabolic rate and insulin sensitivity. Importantly, long-term treatment with high-dose levothyroxine can cause chronic supraphysiological thyroid hormone concentrations (8) with potentially serious adverse effects including cardiac arrhythmias (9). Excessive thyroid hormone has been associated with polyphagia in horses (8) which requires strict dietary restrictions to avoid weight gain during treatment and could pose a compliance issue for owners of ID horses.

Sodium glucose co-transporter 2 inhibitors (SGLT2i) are a relatively new class of drugs that are used in the management of Type 2 diabetes in people. These medications reduce pancreatic insulin secretion by promoting renal excretion of glucose in the urine (10). In veterinary medicine, the SGLT2i velagliflozin has recently become available as the brand name product Senvelgo™ (Boehringer Ingelheim), labeled for the treatment of diabetes in non-insulin-dependent cats. There is no SGLT2i currently available that is labeled for the treatment of hyperinsulinemia in horses; however, equine veterinarians have begun using this class of medications off-label for refractory cases of insulin dysregulation and hyperinsulinemia associated laminitis (HAL) (11). In the authors’ experience, the SGLT2i that is most readily available and economically feasible for use in horses in Canada is canagliflozin. There is some published evidence of the efficacy of canagliflozin for the management of insulin dysregulation and improvement of laminitis in horses (12) and a dose of 0.6 mg/kg given orally once daily has been suggested to be effective at reducing blood insulin concentrations. However, this dose has been associated with elevations in serum triglycerides in treated equines (13) and decreasing the dose to 0.3 mg/kg can reduce its insulin controlling effects (14). There are minimal pharmacokinetic data available to support the establishment of sensible dosing regimens; the sole published work on canagliflozin pharmacokinetics in horses is a single-dose study in 8 animals (15). This study reported a mean half-life of 29.8 ± 1.23 h for canagliflozin in the studied horses. Such a long half-life suggests there may be the potential for accumulation with once daily dosing, and that less frequent dosing might be efficacious and reduce the likelihood of adverse effects. Although relatively few adverse events after canagliflozin administration have been reported in equine patients, in humans this drug can be associated with several important adverse effects including euglycemic ketoacidosis and urinary tract infections (16). Horses are unlikely to develop ketoacidosis due to the relative unimportance of ketogenic pathways to their energy metabolism (17).

## Case description

A 23-year-old university-owned Standardbred mare used for clinical teaching of veterinary students presented with acute, severe (Obel grade 4) laminitis in February 2024 (18). In the following weeks, the mare was treated in hospital and then medically managed (diet, corrective farriery). Despite the mare being in healthy body condition (BCS 4/9), a potential winter-associated exacerbation of insulin dysregulation hyperinsulinemia was retrospectively considered the most likely cause of the episode in the absence of other known contributing factors such as pituitary pars intermedia dysfunction or sepsis-associated or supporting-limb laminitis (19). The mare had consistently normal plasma ACTH levels. A thyrotropin releasing hormone (TRH) stimulation test was not performed due to the lack of availability of TRH in Canada. Markedly high insulin was measured in July 2024 (825 pmol/L; 137.5 μIU/mL) (Reference interval <300 pmol/L; 50 μIU/mL), and insulin was mildly elevated in September 2024 (339 pmol/L; 56.5 μIU/mL). All insulin concentrations were obtained from the University of Guelph’s Animal Health Laboratory (AHL) in Ontario, Canada, using radioimmunoassay (Human Insulin Specific radioimmunoassay, catalog number HI-14 K, MilliporeSigma Canada Ltd., Ontario). The reference interval for insulin was determined based on 39 clinically healthy adult horses that had received no grain for 12 h prior to testing. Results were compared to those of Michigan State University and Cornell University Veterinary Diagnostics labs, using the same methodology (20). Samples for insulin testing were obtained in the early morning prior to any grain feeding; the mare always had free access to hay. Given the patient’s history of severe laminitis and the lack of response to a long-term diet low in non-structural carbohydrates, we elected to treat with the SGLT2i canagliflozin. A course of metformin was not attempted due to the lack of evidence of consistent clinical benefit (5, 6) and the necessity of twice daily administration. In October 2024, following permission from the Veterinary Sciences Animal Care Committee of the University (Protocol AC24-0162), the horse began treatment of hyperinsulinemia to reduce the risk of future HAL episodes. Subsequently pharmacokinetic analysis of plasma canagliflozin concentrations was performed.

At the start of the trial the mare had a body condition score (BCS) of 4/9, weighed 450 kg on a large animal scale (Horse Weigh, United Kingdom), and was measured at 410 kg by weight tape (Supra Height and Weight Tape; Greenhawk Equestrian Sport, Ontario). During the initial phase of the trial the mare was ambulating comfortably. A complete physical examination was performed by the attending veterinarian. No abnormalities were noted except for mild mammary gland edema, which is a noted risk factor for ID (19). Urinalysis, a complete blood count, serum biochemistry, and ACTH were all within normal limits. Lameness score, hoof temperature, and forelimb digital pulses were normal at the start of the trial and were monitored daily during treatment.

The phenotype of this case is consistent with lean-insulin dysregulation (lean-ID), which is potentially more difficult to treat with dietary management because caloric restriction is not indicated (21). In this case, the patient was offered free choice grass hay and was also fed a small amount of low-carbohydrate feed (Step 8, Trouw Nutrition, Canada) to administer the medication, a daily ration balancer (Gro ‘N Win ration balancer, Buckeye Nutrition) and ad libitum water. During summer months, the patient had access to a small amount of grass to accommodate the welfare considerations of keeping her with her equine companion as well as providing an opportunity for her to express foraging behaviors throughout this long study. Due to the dry climate in Alberta, accessible summer grass was never long and/or lush. Increased exercise was not warranted for this case due to the patient’s optimal BCS and history of severe laminitis.

Freely available water was particularly important during this trial because of the potential for osmotic diuresis resulting from glucosuria secondary to SGLT2i administration (22). The excretion of glucose in the urine induced by SGLT2 inhibition reduces insulin production by the pancreatic beta cells (23) and can cause polyuria and polydipsia which has been reported in 19% of horses being medicated with the SGLT2i ertugliflozin (24). Monitoring for polyuria and polydipsia in this case was primarily done in the field by husbandry care staff, or by study staff when the horse was stabled during participation in teaching labs or for urine collection; water intake and urination remained subjectively normal throughout the trial. In humans, adverse effects of treatment with SGLT2i can include urinary tract and genital infections (due to increased glucose excretion in the urine) and, although not currently reported in equine literature (14, 24, 25), the patient was monitored for these conditions. Hypertriglyceridemia of varying severity is a common sequalae of SGLT2i treatment in equines (12, 13, 24) and therefore serum triglycerides were measured weekly. In addition, the patient was monitored daily for dullness, signs of colic, and anorexia. Euglycemic ketoacidosis (due to negative energy balance) is another potential adverse effect of SGLT2i treatment in humans and cats but is unlikely in horses (17). No adverse effects of canagliflozin treatment were observed throughout the trial.

### Treatment regimens, patient monitoring, and pharmacokinetic testing

Our objectives were to treat the mare’s ID to reduce the risk of future HAL episodes, and to obtain pharmacokinetic data for single, daily, and EOD administration of canagliflozin to provide the first evidence of canagliflozin accumulation, or lack thereof, on standard dosing regimens. A dose of approximately 0.6 mg/kg canagliflozin (one Invokana 300 mg tablet per dose) was chosen as recommended in current literature to reduce insulin levels and improve hoof comfort (14). This was administered in a handful of feed that is low in non-structural carbohydrates (Step 8, Trouw Nutrition, Canada) and hay was not withheld at any point during the treatment.

**Figure 1:**
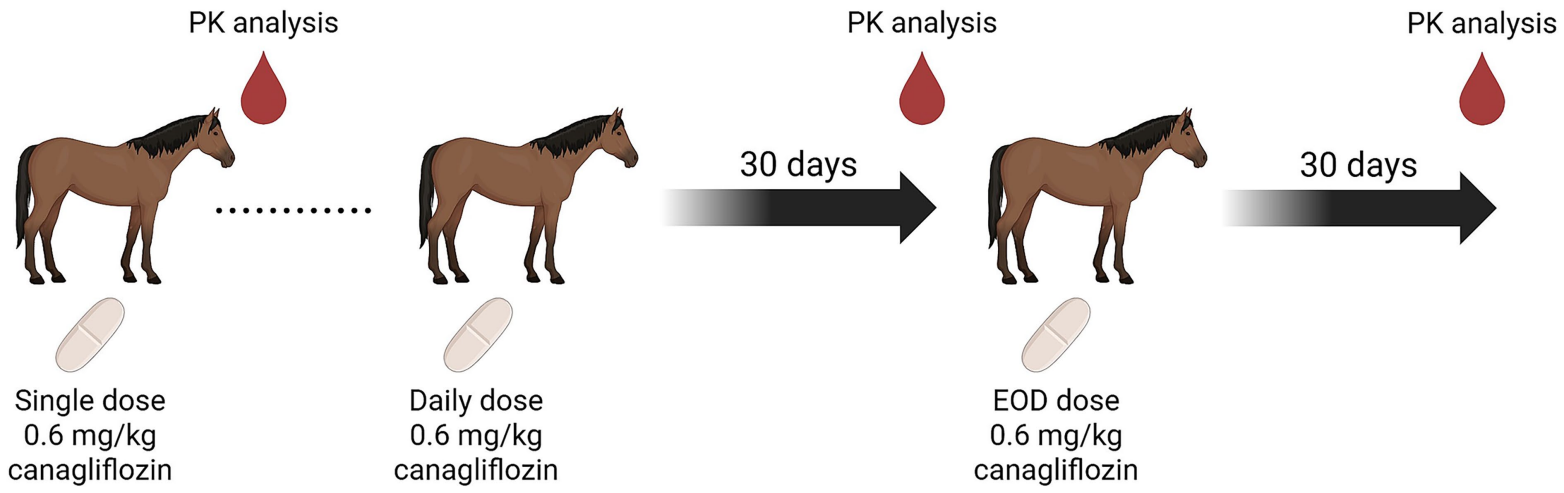
Timeline of canagliflozin administration and plasma sampling for pharmacokinetic analysis. EOD, every other day. Created in BioRender. Rosa, B. (2026) https://BioRender.com/k1cbrj6.

**Figure 2:**
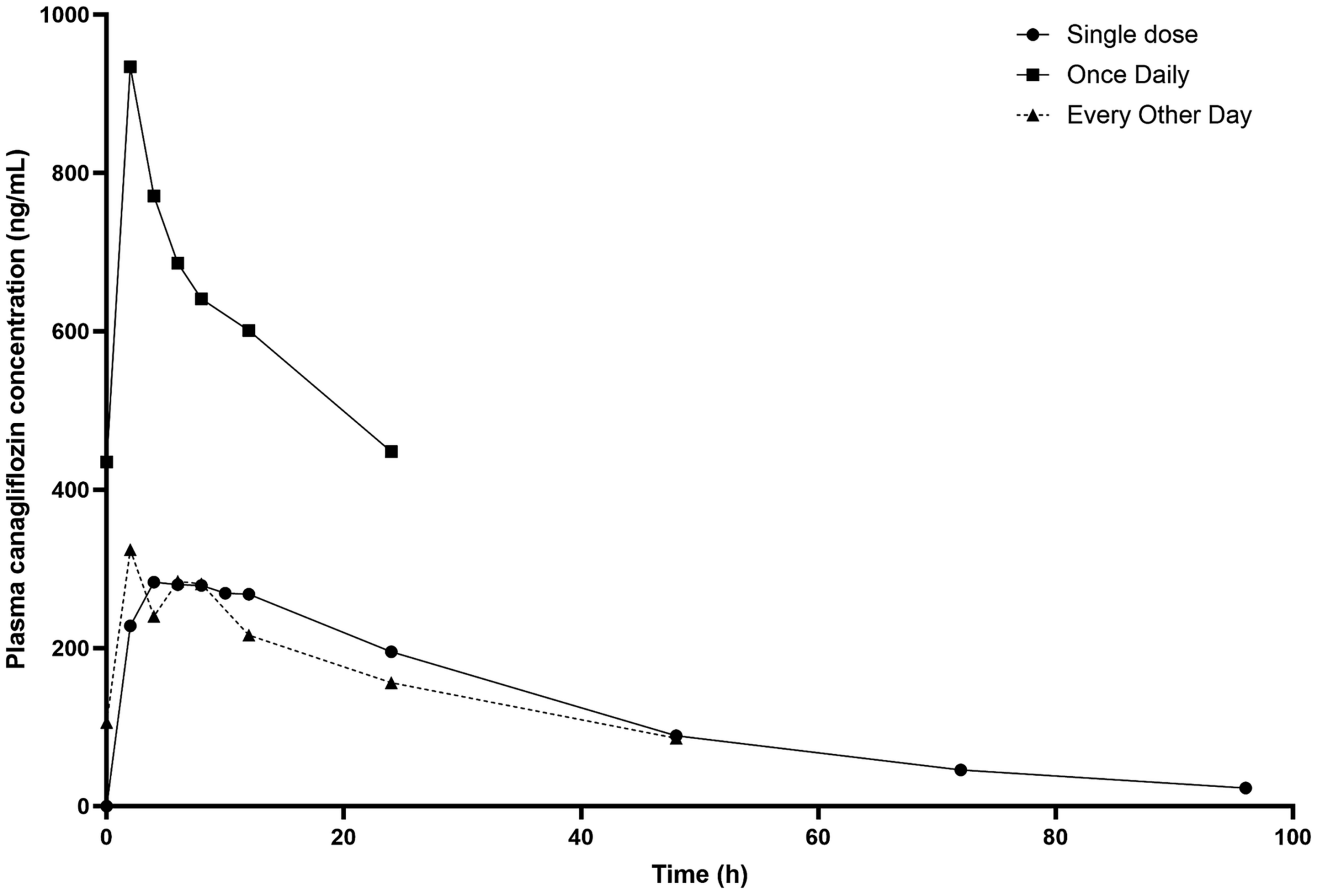
Pharmacokinetics of canagliflozin after different dosing regimens in a single horse.

A schematic illustration and timeline of the treatment and sampling protocol is shown in Figure 1. All blood samples for pharmacokinetic analysis were obtained via intravenous catheter for hours 0 through 12, and then by direct jugular venipuncture. Blood was collected into sodium heparin tubes (Becton Dickinson, United States) and plasma was separated immediately after collection and then stored at −80 °C until analysis. After single dose administration, plasma samples were obtained at baseline and at 2, 4, 6, 8, 10, 12, 24, 48, 72, and 96 h. Our initial plan to start daily treatment 1 week following single dose data collection was delayed because an insulin test submitted prior to drug administration came back within normal limits (186 pmol/L; 31 μIU/mL) and the horse was clinically stable. Serum insulin 24 h after single dose administration was elevated at 314 pmol/L (52.3 μIU/mL), but since the value one day prior had been normal and the mare was asymptomatic this was attributed to a possible rebound effect after administration due to compensatory glucose uptake by sodium-glucose cotransporter 1 (SGLT1) in the proximal tubule of the kidney, which has been shown in mice to occur rapidly after pharmacological inhibition of SGLT2 (26). Other possibilities for this result include a sampling error such as accidental feeding or lab error. Further treatments were not started at this time. However, in May 2025 the mare experienced a moderate laminitis flare-up, serum insulin levels were 381 pmol/L (63.5 μIU/mL), and the decision was made to proceed with the trial. Canagliflozin was administered once daily for 30 days, after 30 days of daily treatment heparinized plasma was collected immediately before dosing and at 2, 4, 6, 8, 12, and 24 h after administration. At 48 h after the final once-daily dose, every other day (EOD) dosing commenced. After 30 days of EOD treatment, sampling was repeated immediately before medication administration and at 2, 4, 6, 8, 12, 24, and 48 h. Figure 2 shows the time-plasma concentration curves after single dose, once daily, and EOD administration. The mare was doing well and continued EOD treatment for an additional 2 months. As she continued to do very well, we attempted to discontinue treatment, slowly tapering canagliflozin over a period of 4 weeks, administering a dose every 3 days for 5 doses, followed by a dose every 4 days for 5 doses and then stopping the medication. Near the end of her weaning period, her insulin level was within normal limits at 113 pmol/L (18.8 μIU/mL); however, 4 weeks after treatment stopped, she appeared mildly footsore and routine monitoring revealed that her insulin was 501 pmol/L (83.5 μIU/mL). The insulin concentration in a post-prandial sample taken in the same time period was 1,153 pmol/L (192.2 μIU/mL). Given her previous laminitis history, EOD canagliflozin was restarted and she has remained comfortable and normo-insulinemic on this treatment.

**Figure 3:**
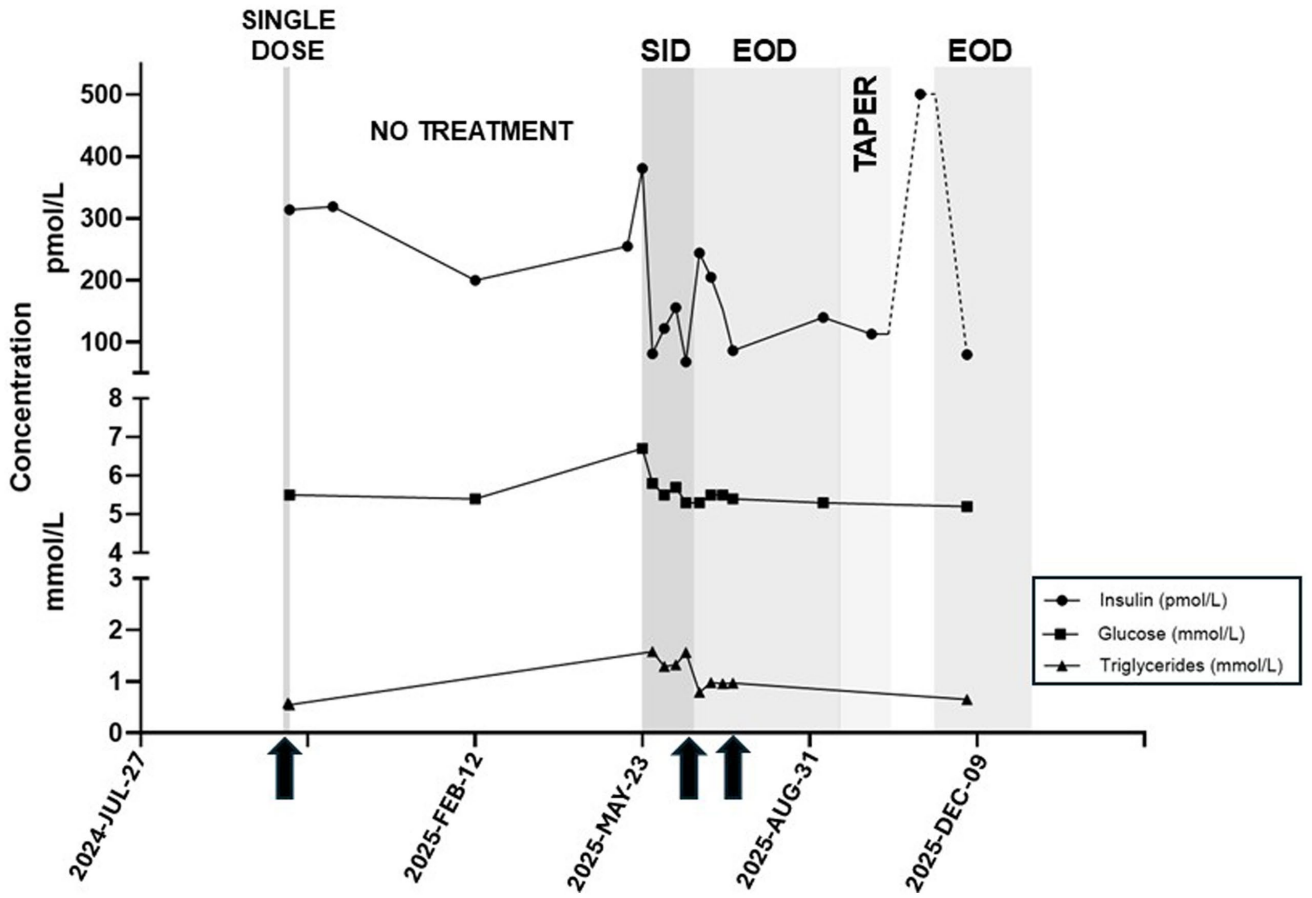
Serum insulin, glucose, and triglyceride concentrations relative to canagliflozin administration using 3 different dosing protocols: single dose, once daily (SID), and every other day (EOD). Insulin concentrations are expressed in pmol/L. To convert to μIU/mL divide the pmol/L value by 6.0. Glucose and triglycerides are expressed in mmol/L. The solid black arrows indicate the timing of sample collection for pharmacokinetic analysis, which was performed after 1 month of treatment for both the single dose and EOD protocols.

During treatment, serum insulin and glucose, were monitored weekly by submission to the Animal Health Laboratory, University of Guelph, Canada and triglyceride concentrations were monitored weekly by submission to Idexx, Calgary, Alberta, Canada (Figure 3). Samples were collected first thing in the morning after only hay feeding overnight according to current laboratory recommendations. Serum samples were allowed to clot for approximately 30 min prior to being centrifuged at 1000–2000 x g for 10 min using an LW Scientific E8 centrifuge, separated, and frozen.

The pharmacokinetic parameters for canagliflozin in this patient, generated by noncompartmental analysis using PKsolver (27) are shown in Table 1. Plasma canagliflozin concentrations were obtained by liquid chromatography–tandem mass spectrometry (Vogon Laboratories, Cochrane, AB). The elimination half-life after single dose administration in this patient was similar to previously reported values (15) and the plasma canagliflozin concentration was 23 ng/mL at 96 h after administration. Daily dosing led to accumulation and the accumulation ratio (AUC_0-inf_ multidose/AUC_0-inf_ single dose) was 2.73. Dosing EOD eliminated accumulation and the accumulation ratio after EOD treatment was 0.93. The predicted steady state concentration (C_ss_) for daily dosing calculated from our single dose data using the formula C_ss_ = AUC_0-inf_ / dosing interval is 518 ng/mL. This is consistent with what we observed during once daily administration (Figure 2).

**Table 1:** Pharmacokinetic parameters for 300 mg canagliflozin in a 450 kg horse after a single dose, once daily dosing for 1 month, and every other day (EOD) dosing for 1 month.

Treatment	C_max_ (ng/mL)	T_max_ (h)	AUC _0-t_ (ng/mL*h)	AUC _0-inf_ (ng/mL*h)	T_1/2_ (h)	MRT (h)
Single dose	283.0	4	11582.9	12424.5	24.9	35.6
Daily dosing	934.2	2	14633.1	33897.2	29.8	42.7
EOD dosing	324.3	2	8219.7	11599.8	27.2	38.5

Noncompartmental pharmacokinetic analysis was performed using PKsolver (27). C_max_, maximum concentration; T_max_, time to maximum concentration; T_1/2_, terminal half-life; AUC_0-t_, area under the plasma-concentration-time curve from time zero to last measured concentration (at 96h for single dose, 24h for daily dosing, and 48h for EOD dosing); AUC_0-inf_, area under the plasma concentration-time curve extrapolated from time zero to infinity; MRT, mean residence time.

Reports of ertugliflozin in humans show rapid absorption following oral administration (1–2 h post dose) (28). In our patient, the maximum measured plasma concentration (Cmax) was 4 h after a single dose and at 2 h following daily or EOD dosing. This was faster than previously reported (15), however the horses in the previous study were given a higher dose of canagliflozin (1.8 mg/kg). Note that the 48-h blood sample obtained after 30 days of daily dosing was omitted from analysis because the medication had been accidentally administered 20 min prior to blood sampling and a rise in plasma canagliflozin concentration was already evident, confirming that some absorption occurs almost immediately following oral administration. When comparing our data to that of Michanek et al. (15) who reported canagliflozin pharmacokinetics in 8 Icelandic horses after a single oral dose of 1.8 mg/kg, the Cmax and AUC that we obtained, and the C_ss_ that we predicted for daily dosing, are lower than would have been expected under the assumption of linear kinetics. However, there is considerable interindividual variability in canagliflozin pharmacokinetics (15) and this highlights the need for more studies in a larger population of horses. These values may also have been affected by the impact of fed-status on canagliflozin absorption. The horses in the Michanek study were fasted for 1 h before and 8 hrs after drug administration, while our horse had free access to hay. Although in humans there is no clinically relevant effect of food on canagliflozin absorption (31), there are no data on the impacts of fed-status on the absorption of canagliflozin in horses.

Previous studies have reported an increase in serum triglyceride concentrations with SGLT2i treatment (12, 24). Similar results were seen in this case with an initial peak at 1.58 mmol/L (reference range 0.11–1.02 mmol/L) after commencing daily dosing of canagliflozin. Although triglyceride concentrations were reported to have increased in previous case studies, it was observed that they eventually declined with daily dosing and typically did not result in clinical signs of hyperlipemia (24, 29). Due to the short duration of once daily dosing prior to switching to every other day dosing, it cannot be confirmed whether the eventual decrease in triglycerides seen with this case was due to the change in dosing or a similar decline seen in previous once daily dosing studies. No clinical signs of hyperlipemia were observed, and triglyceride concentrations returned to within normal reference intervals within 60 days of starting regular treatment. Evidence of hepatic lipidosis and hypoglycemia was not observed throughout the treatment period. Serum chemistry samples were taken approximately 3 weeks into daily and EOD treatment, and all values were within normal limits.

Polyuria and polydipsia were not observed throughout this case study and obtaining a free-catch urine sample for analysis was difficult. Attempts were made weekly to collect a free catch urine sample and, when successful, urinalysis was performed using Jorvet Vet-10 reagent strips. As expected, and as reported in other literature (14), urine glucose was found to be approximately 55 (+++) mmol/L during daily treatment. During EOD treatment urine glucose ranged from approximately 28 (++) to 55 (+++) mmol/L. This slight apparent semiquantitative reduction in glucosuria seen with EOD treatment may indicate that less frequent canagliflozin dosing resulted in less excretion of glucose in the urine; however, urine samples were difficult to collect and our sparse sampling does not allow a robust comparison of urine glucose between treatment regimens. A urine sample obtained Oct 20th, 2025, 3 days following weaning the horse off of canagliflozin completely, still contained approximately 28 to 55 mmol/L urine glucose, which suggests there was still some clinically relevant SGLT2i activity at this time. The efficacy of an every third day dosing regimen is beyond the scope of this case report but should be considered. Ketones, leukocytes, and blood parameters were consistently negative, and other clinical signs consistent with a urinary tract infection were not observed.

Access to a scale was only available at the beginning of this case study. Body weight was noted to be 450 kg by scale and 410 kg by weight tape and BCS was 4/9 in May 2025. Weight loss during SGLT2i treatment in horses is reported (estimated 6–37 kg/30-day treatment (30)). For this case, BCS and weight tape measurements stayed consistent until September 2025 but subtle changes in weight may have been missed due to the lack of sensitivity of using an equine weight tape or the BCS scoring system. In September 2025, the mare was noted to appear thinner (BCS = 3/9) despite consistent weight tape measurements (402 kg). This cannot definitively be attributed to SGLT2i treatment as dental examination revealed a molar which required extraction and this may have contributed to her weight loss during the weaning stage of treatment.

The patient in this study had previously suffered severe laminitis but was only moderately lame at the commencement of the daily treatment regimen (May 2025). It is difficult to determine if the rapid improvement of lameness at this time was solely or partially due to the canagliflozin treatment or the result of other factors. Nevertheless, the patient has not suffered a laminitis flare-up at any time during treatment.

From a client perspective successfully managing hyperinsulinemia using EOD treatment reduces medication costs. Although further investigation is required, EOD treatment may also result in a lower incidence of medication-associated adverse effects and may also lessen the required frequency and associated costs of veterinary oversight during treatment, hopefully improving access to care for the horse and pony population. Our patient maintained low serum insulin levels while being weaned off the drug but rapidly reverted to hyperinsulinemia once treatment was stopped completely, suggesting that some horses may be able to achieve insulin control with less frequent dosing than EOD, although this warrants further investigation.

This case report has several important limitations. Serum insulin was not measured during the initial episode of severe laminitis, so the diagnosis of HAL was made retrospectively when it was recognized that the horse was chronically hyperinsulinemic. Pharmacokinetic data is only provided over a single dose for each treatment regimen; therefore, intra-individual variability is not accounted for in our findings. As expected with a case report, we are only able to report our experiences with one lean-ID patient; thus, the generalizability of these findings to the wider equine population, specifically overweight and acutely laminitic patients, requires further investigation. Despite these limitations, this is the first report of successful control of insulin in a horse with ID using canagliflozin with an EOD dosing protocol. In this lean-ID patient, insulin levels were maintained within normal limits with EOD oral canagliflozin at a dose of 0.6 mg/kg and the horse remained comfortable. Due to the long terminal half-life of canagliflozin, daily dosing of canagliflozin resulted in accumulation and approximately three times the drug exposure achieved with EOD dosing. In addition, EOD dosing was associated with lower triglyceride levels than daily dosing, potentially reducing the likelihood of drug-related adverse effects. Lowering insulin levels while also reducing potentially harmful side effects, such as hypertriglyceridemia (11, 13), may make EOD treatment the optimal dosing regimen for horses. However, inadequately controlled hyperinsulinemia can result in additional laminitis episodes. Therefore, prospective studies investigating the incidence of laminitis and SGLT2i-associated adverse events in risk-matched populations are needed to understand the optimal dosing regimen for horses. The pharmacokinetic data included in this case report provide some pharmacological context for the observed clinical effects of canagliflozin and can serve as a basis for designing future, larger studies.

### Patient perspective

The patient in this study is a valuable member of the University teaching herd, and she continued her teaching role during and after this trial. As is the goal with all patients, her comfort and continued amenability to handling and veterinary procedures was of paramount importance. Throughout treatment, the medication was given in a small amount of feed and not syringed, reducing the likelihood of the patient developing head shyness. All blood sampling and intravenous catheterizations were performed by experienced veterinary personnel or veterinary students under supervision of experienced veterinary personnel. Intravenous catheterizations were performed through a lidocaine bleb to reduce sensation of the large bore catheter. The improved understanding of canagliflozin pharmacokinetics that we have achieved through this trial continues to help us manage this patient, and other insulin dysregulated members of our teaching herd, successfully on an EOD dosing regimen.

## Data Availability

The original contributions presented in the study are included in the article, further inquiries can be directed to the corresponding author.
